# Nelfinavir, an HIV-1 Protease Inhibitor, Induces Oxidative Stress–Mediated, Caspase-Independent Apoptosis in *Leishmania* Amastigotes

**DOI:** 10.1371/journal.pntd.0000642

**Published:** 2010-03-30

**Authors:** Pranav Kumar, Robert Lodge, Nathalie Trudel, Michel Ouellet, Marc Ouellette, Michel J. Tremblay

**Affiliations:** Centre de Recherche en Infectiologie, Centre Hospitalier de l'Université Laval, and Département de Microbiologie et Immunologie, Université Laval, Québec, Canada; AP-HP, service de parasitologie-mycologie, France

## Abstract

**Background:**

Visceral leishmaniasis has now emerged as an important opportunistic disease in patients coinfected with human immunodeficiency virus type-1 (HIV-1). Although the effectiveness of HIV-1 protease inhibitors, such as nelfinavir, in antiretroviral therapies is well documented, little is known of the impact of these drugs on *Leishmania* in coinfected individuals.

**Methodology and Principal Findings:**

Here, we show that nelfinavir generates oxidative stress in the parasite, leading to altered physiological parameters such as an increase in the sub-G1 DNA content, nuclear DNA fragmentation and loss of mitochondrial potential, which are all characteristics of apoptosis. Pretreatment of axenic amastigotes with the caspase inhibitor z-VAD-fmk did not inhibit the increase in sub-G1 DNA content in nelfinavir-treated parasites, suggesting therefore that this antiviral agent does not kill *Leishmania* amastigotes in a caspase-dependent manner. Furthermore, we observed that the mitochondrial resident protein endonuclease G is involved. We also demonstrate that parasites overexpressing GSH1 (the rate limiting enzyme of glutathione biosynthesis) were more resistant to nelfinavir when compared to untransfected controls.

**Conclusions and Significance:**

These data suggest that nelfinavir induces oxidative stress in *Leishmania* amastigotes, culminating in caspase-independent apoptosis, in which DNA is degraded by endonuclease G. This study provides a rationale for future, long-term design of new therapeutic strategies to test nelfinavir as a potential antileishmanial agent as well as for possible future use in *Leishmania*/HIV-1 coinfections.

## Author Summary

Visceral leishmaniasis is the most severe form of disease caused by the parasite *Leishmania*. It is a major concern in South America, Africa, India and the Middle East. Additionally, it has now emerged as an important opportunistic disease in patients coinfected with HIV-1. This is due, in part, to the increasing overlap between urban centers and rural areas endemic for *Leishmania*. Although more efficient combinatorial antiviral drug regimens for treating HIV-1 infection have been developed, the impact of such therapies on HIV-1/*Leishmania* coinfection is yet to be explored. In this study, we investigated the effect of nelfinavir, a well-characterized anti-HIV-1 drug, on *Leishmania*. Treating the parasite with nelfinavir activates events that are hallmarks of programmed cell death (also called apoptosis). Among these are oxidative stress, changes in DNA replication and fragmentation, and release of mitochondrial enzymes. Furthermore, these events occur without the participation of caspases, which are classically linked to apoptosis; however, this atypical apoptosis requires the translocation of endonuclease G from mitochondria to the cytoplasm. These findings provide insights for the design of new anti-parasitic therapies, particularly in the case of *Leishmania*/HIV-1 coinfections.

## Introduction

*Leishmania* is a family of zoonotic protozoan parasites that cause a variety of human diseases termed leishmaniases, which have a current annual incidence of 2 million new cases in 88 countries. Depending on the species involved, infections in humans may lead to a broad spectrum of clinical manifestations, ranging from ulcerous skin lesions to more serious and potentially fatal visceral diseases. Mobile, flagellate *Leishmania* promastigotes propagate in the sand fly vector, and eventually differentiate into non dividing metacyclic forms before inoculation into the vertebrate host and phagocytosis by macrophages. The metacyclics subsequently differentiate into amastigotes, which multiply in an intracellular vacuolar compartment, leading to macrophage lysis and serial infection of other surrounding macrophages. Besides being a major tropical disease, leishmaniasis, and particularly visceral leishmaniasis (VL) which is caused primarily by *Leishmania infantum* (*L. infantum*) and *L. donovani*, is now also emerging as an important opportunistic disease found in patients infected with human immunodeficiency virus type-1 (HIV-1) [1]. Indeed, reactivation of latent *Leishmania* infections have been reported to occur frequently in HIV-1-infected and immunocompromised individuals [2],[3], and, on the other hand, the parasite is able to potentiate and up-regulate virus replication, at least *in vitro* [4],[5].

The recent development of highly active antiretroviral therapy (HAART) has significantly improved the prognosis of patients infected with HIV-1. This therapeutic strategy consists of at least three anti-retroviral drugs, typically two nucleoside or nucleotide reverse transcriptase inhibitors (NRTIs) used in combination with a non-nucleoside reverse transcriptase inhibitor (NNRTI) or a protease inhibitor (PI). It has been demonstrated that PIs block the active site of aspartyl protease, a viral enzyme known to be essential for the maturation of viral proteins, by mimicking peptides [6]. In patients co-infected with *Leishmania* and HIV-1, HAART is initiated to partially restore immune functions and it has also been found to prevent VL in individuals infected by *Leishmania* [7], as reflected by the sharp decrease in the incidence of VL in Europe following the widespread use of HAART [8],[9]. Recently, two HIV-1 PIs (i.e. indinavir/IDV and saquinavir/SQV) were described to exert a dose-dependent antileishmanial activity against *L. infantum* and *L. major* promastigotes *in vitro* [10]. Interestingly, we have recently reported the leishmaniacidal activity of PIs in an experimental cell culture system, in which we show that nelfinavir (NFV) acts as a powerful *in vitro* inhibitor of the intracellular growth of *Leishmania* in both monocytoid THP-1 cells and human primary monocyte-derived macrophages [11]. Moreover, subcytotoxic concentrations of NFV and ritonavir (RTV) significantly inhibit the growth of axenic amastigotes *in vitro* [11]. Although these previous studies clearly show that HIV-1 PIs can inhibit the growth of *Leishmania* parasites, the exact mode(s) of action of such compounds remains to be established. In mammalian cancer cell lines, NFV is known to induce apoptotic death [12]. Interestingly, it was recently demonstrated that a 24 h treatment with the PI lopinavir (LPV) causes chromatin condensation in *L. amazonensis* promastigotes, which is suggestive of apoptosis [13]. However, whether the PI NFV can bring about similar effects on *Leishmania* amastigotes remains unknown.

Previous studies have revealed that apoptosis, which is considered as a tightly regulated form of cell death, plays a dominant role in the interaction between *Leishmania* and its host. For example, primed macrophages were reported to kill intracellular parasites by inducing apoptosis through a caspase- and cysteine protease-independent mode [14],[15]. *Leishmania* can undergo apoptosis under *in vitro* conditions following exposure to various stimuli such as generation of reactive oxygen species (ROS), which will eventually lead to changes in mitochondrial membrane potential, endonuclease activation and DNA laddering. Together these metabolic processes will result in nuclear condensation, cell cycle arrest and surface binding of annexin V to phosphatidylserines. Furthermore, several reports have suggested that *Leishmania* can undergo apoptosis by caspase activation [16],[17],[18],[19]. However no caspase gene has been identified in the *L. major* genome sequence database [20]. Recently, several groups have suggested that a mitochondrial nuclease homologous to endonuclease G (EndoG) is involved in a caspase-independent apoptosis in *Trypanosoma* and *Leishmania* parasites [21],[22],[23]. Moreover, involvement of EndoG in nucleosomal DNA degradation was also found in sustained endogeneous oxidative stress [21],[22],[24]. It is therefore quite obvious that apoptosis of *Leishmania* can be induced by distinct pathways [16],[18],[19],[22], although it is unclear if any of these mechanisms are involved in the previously reported NFV-mediated antileishmanial activity [11].

In the present study, we report that NFV induces apoptosis in axenically grown *Leishmania* amastigotes. Moreover, we show that NFV mediates a caspase-independent apoptosis via an oxidative stress, which culminates in mitochondrial membrane depolarization in the protozoan parasite *Leishmania*. This biochemical event leads to a release of EndoG from the depolarized mitochondria resulting ultimately in DNA fragmentation. Together our results provide the first insights into the cell death mechanisms induced by NFV in the *Leishmania* parasite.

## Materials and Methods

### Chemicals

The HIV-1 PI NFV was obtained from the NIH AIDS Repository Reagent Program (Germantown, MD). This compound was resuspended to a concentration of 50 mM in dimethyl sulfoxide (DMSO) for a maximum final concentration in culture of 0.1% DMSO. The benzyloxycarbonyl-Val-Ala-DL-Asp(O-methyl)-fluoromethylketone (z-VAD-fmk) reagent, a caspase inhibitor with broad specificity, was purchased from Sigma-Aldrich (St. Louis, MO), was dissolved in DMSO at 20 mM concentration and stored at −20°C. Aurintricarboxylic acid ammonium salt (ATA, 20 mM, stock solution, Sigma-Aldrich) and N-Acetyl cysteine (NAC, 0.5 M, stock solution, Sigma-Aldrich) were dissolved in water and stored at −20°C. Propidium iodide (Sigma-Aldrich) was also dissolved in water at a final concentration of 1 mg/ml and stored at 4°C. Miltefosine was from Cayman Chemicals (Ann Arbor, MI) and stored at −20°C.

### Parasite culture and maintenance

Axenically grown amastigotes of *L. donovani* field strain 9518 (Ld 9518) were maintained at 37°C with 5% CO_2_ by weekly subpassages in MAA/20 medium pH (5.6) in 25-cm^2^ flasks. MAA/20 consists of modified medium 199 (Gibco BRL, Carlsbad, CA) with Hank's salts, supplemented with 0.5% soybean trypto-casein (Pasteur Diagnostics, Marne la Coquette, France), 15 mM D-glucose, 5 mM L-glutamine, 4 mM NaHCO_3_, 0.023 mM bovine hemin and 25 mM HEPES at a final pH of 6.5 and supplemented with 20% of fetal bovine serum (FBS). These axenic amastigotes show morphological, biochemical and biological characteristics similar to those of amastigotes isolated *in vivo*. *L. infantum* (MHOM/MA/67/ITMAP-263) promastigotes transfected with pSPαzeoα and pSPαzeoαGSH1, hereafter called Li-pSP and Li-GSH1, were maintained at pH 7.0 and at a temperature of 25°C and cultured in RPMI-1640 medium (Wisent, St.-Bruno, Quebec) supplemented with 10% FBS, 5 µg/mL hemin, 25 µM HEPES, 2 mM NaHCO_3_ and 800 µg/ml of the selective agent Zeocin (Invivogen, San Diego, CA) in 25-cm^2^ flasks. Li-pSP and Li-GSH1 axenic amastigotes were obtained after several days of culture by seeding promastigotes in 25-cm^2^ flasks containing MAA/20 medium at a final pH of 5.6 as described above except for the addition of 800 µg/ml Zeocin.

### Selection of NFV-resistant amastigotes

Axenically grown amastigotes of *L. donovani* Ld 9518 were subjected to stepwise increasing drug concentrations until resistance to 6.25 and 12.5 µM of NFV was established. A stepwise increase in drug concentration was undertaken only when the drug-exposed cultures showed a growth rate equivalent to that of unexposed cultures. Parasites displaying a resistance phenotype were maintained under a constant drug pressure (i.e. 6.25 and 12.5 µM) throughout our entire experimental procedures. Resistant clones were also stored in −150°C at various times during their establishment.

### DNA fragmentation assays

Log-phase axenic amastigotes were seeded at a final concentration of 5×10^6^ parasites/ml and drugs were added to the culture media at the listed concentrations. Total DNA (i.e. 10 µg) was extracted at different intervals by the salting out method [25]. Parasites were then pelleted and resuspended in 300 µl of lysis buffer (i.e. 10 mM Tris-HCl pH 8, 5 mM EDTA, 0.5% SDS, 200 mM NaCl and 100 µg/ml proteinase K) for 45 min at 65°C. Two volumes of ice-cold ethanol (100%) were added and DNA was collected by centrifuging at 13,000 rpm for 15 min in a microfuge. Supernatants were discarded and dried pellets of DNA were resuspended in 50 µl of 10 mM Tris-HCl pH 8 and 0.1 mM EDTA, and treated with RNase A (0.3 µg/ml) for 1 h at 37°C. Qualitative analysis of nuclear DNA fragmentation was performed by 1% agarose gel electrophoresis. In some studies, untreated or NFV-treated axenic amastigotes were washed in PBS. Pelleted cells were permealized in hypotonic lysis buffer (0.1% sodium citrate and 0.1% Triton X-100) and incubated overnight at 4°C containing 50 µg/ml propidium iodide and 100 µg/ml RNase. The fluorescence intensity of propidium iodide was analyzed with a Coulter EPICS XL flow cytometer (Beckman, Miami, FL) and FCS express software (version 3).

### Measurement of Δψ_m_

Change in Δψ_m_ was measured by Cayman's JC-1 mitochondrial membrane potential assay kit (Cayman Chemical Company, Ann Arbor, MI). This dye accumulates in the mitochondrial matrix under the influence of Δψ_m_ and its monomeric form increases in unhealthy or apoptotic cells [26]. Ld 9518 amastigotes were cultured in 24 well plates at a density of 5×10^5^ cells/well in 400 µl of MAA/20 medium in a CO_2_ incubator overnight at 37°C. Parasites were then treated with 40 µM NFV for different time periods afterwhich 40 µl of JC-1 staining solution (prepared according to the manufacturer's instructions) was added to each well. The cells were then incubated at 37°C in a 5% CO_2_ incubator for 30 min, and transferred to 96-well black culture plates. Finally, a spectrofluorometer using 485 nm and 535 nm as excitation and emission wavelengths, respectively, was used to measure Δψ_m_.

### Cell fractionation and immunoblotting

Ld 9518 axenic amastigotes (5×10^6^ cells/ml) were treated with 40 µM of NFV for different time periods before monitoring the cytoplasmic translocation of EndoG. In some studies, parasites were first treated with NAC (20 mM) prior to exposure to NFV. Cell fractionation was carried out using the ApoAlert Cell fractionation kit (Clontech, Mountain View, CA). After the separation of cytosolic and mitochondrial fractions, 5 µg of protein from each fraction was electrophoresed in different sets of 12% SDS-PAGE and transferred to nitrocellulose membranes. Thereafter, membranes were blocked using 5% skimmed milk and immunoblotted with rabbit polyclonal anti-human EndoG (Santa Cruz Biotech, Santa Cruz, CA), followed by anti-rabbit HRP-conjugated antibodies. For loading controls corresponding to mitochondrial and cytosolic fractions, the same blots were reprobed with anti-COX IV antibodies provided with the ApoAlert Cell fractionation kit and anti-human DHFR antibodies (Santa Cruz Biotech) followed by the appropriate HRP-conjugated secondary antibodies. The anti-EndoG, anti-COX IV and anti-DHFR antibodies show cross-reactivity with the corresponding *Leishmania* proteins.

### Statistical analysis

The statistical significance of the results was defined by performing a 1-way analysis of variance with Bonferroni's multiple comparison tests and unpaired t test. All statistical analyses were performed using Prism software 3.03. *P* values of less than 0.05 were considered statistically significant.

## Results

### NFV induces an apoptosis-like death in *Leishmania*

Our initial studies were performed with the 9551 field strain of *L. donovani*. However, we were unable to obtain axenic amastigotes from this viscerotropic strain of *Leishmania*. Therefore, we used the sodium stibogluconate (SbV)-resistant *L. donovani* field strain 9518 (Ld 9518) in the majority of experiments described in the present work, of which we regularly could obtain axenic amastigotes in our laboratory. It should be noted that this *L. donovani* field isolate was previously shown to be sensitive to NFV in promastigote-infected human primary monocyte-derived macrophages [11].

**Figure 1:**
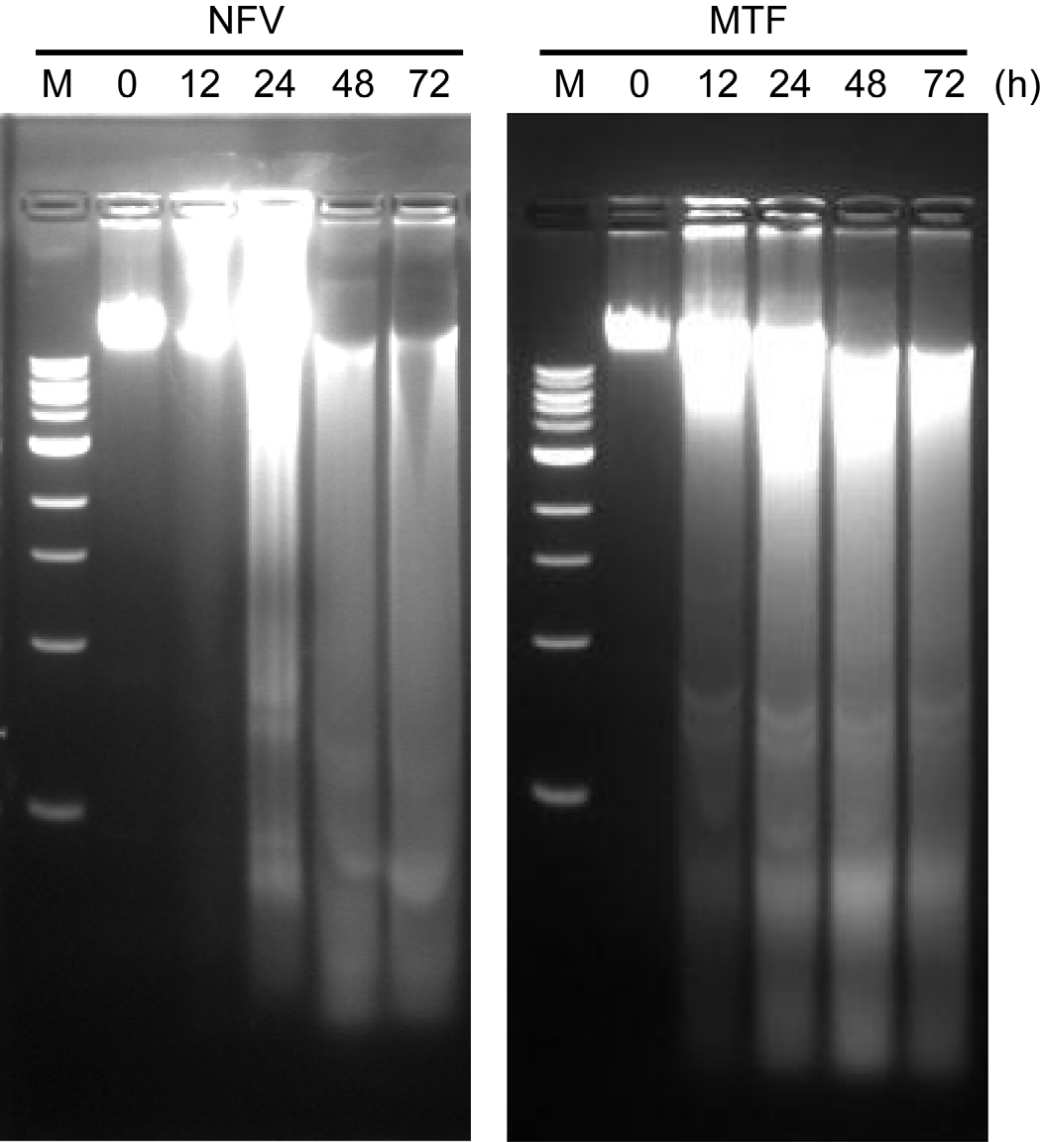
NFV induces degradation of genomic DNA isolated from *L. donovani* amastigotes. Genomic DNA was isolated from Ld 9518 amastigotes which have been treated with NFV or MTF (25 µM in both instances) for the indicated time periods and was resolved on 1% agarose gel. M corresponds to 1 Kb DNA ladder.

In order to investigate the mechanism(s) by which NFV affects *Leishmania* growth, we analysed the integrity of nuclear DNA of untreated and NFV-treated Ld 9518 amastigotes by agarose gel electrophoresis. A combination of ladder and degradation patterns of nuclear DNA fragments was observed beginning at 24 h of NFV treatment, although more pronounced degradation of DNA was achieved after longer treatment (Fig. 1). Miltefosine (MTF) was used as a positive control in this study, having been already shown to induce apoptosis in *Leishmania* [27],[28],[29]. Therefore, as expected, a comparable degradation of nuclear DNA fragments was also observed after a 12 h treatment with MTF, culminating to complete degradation after 24 h of treatment. DNA of untreated parasites, or that of parasites treated with the highest concentration of diluent (i.e. 0.05% DMSO), showed no fragmentation (data not shown).

**Figure 2:**
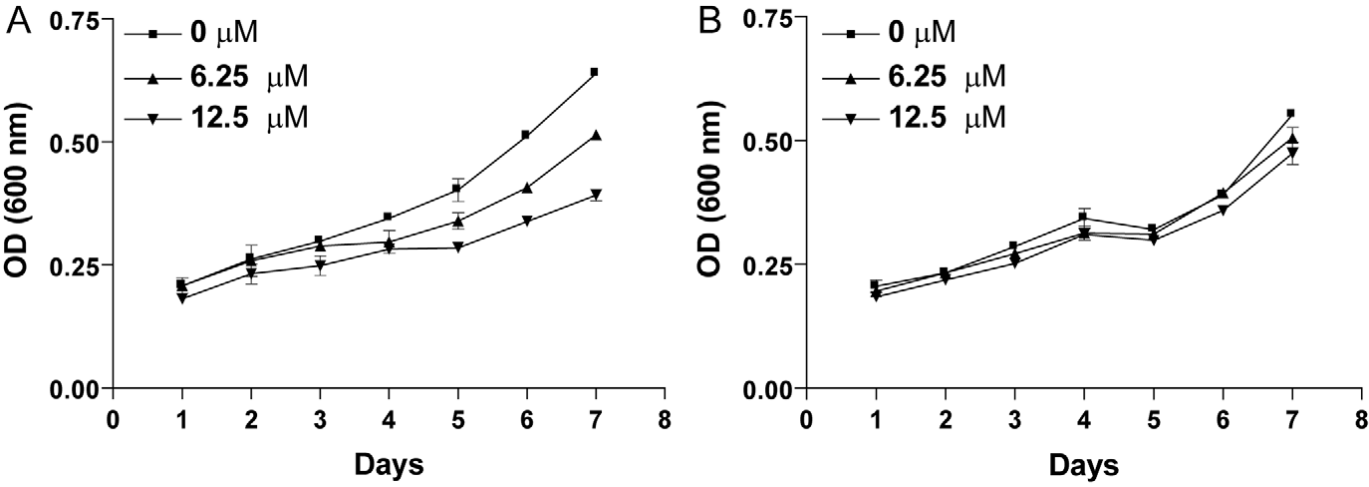
Development of *in vitro* NFV resistance by direct drug pressure on axenic amastigotes. (A) Growth curve was determined at the initial seeding. (B) Growth curve was determined after 8 passages. The growth of parasites was followed by measuring the optical density (OD) at 600 nm at the listed days.

To investigate more deeply the effect of NFV on *Leishmania*, we generated NFV-resistant mutants by increasing the drug concentration in several steps, as previously described [30]. Parasites were continuously maintained under drug pressure and optical density was measured at every passage. After 7 days of culture, we observed a 21% and 33% decrease in parasite growth when using NFV at 6.25 and 12.5 µM, respectively (*P*<0.001) (Fig. 2A). After 8 consecutive passages, the growth rates of parasites treated with the two tested concentrations of NFV were similar to the untreated parasites (Fig. 2B), thus suggesting that we are able to derive parasites displaying a resistant phenotype.

**Figure 3:**
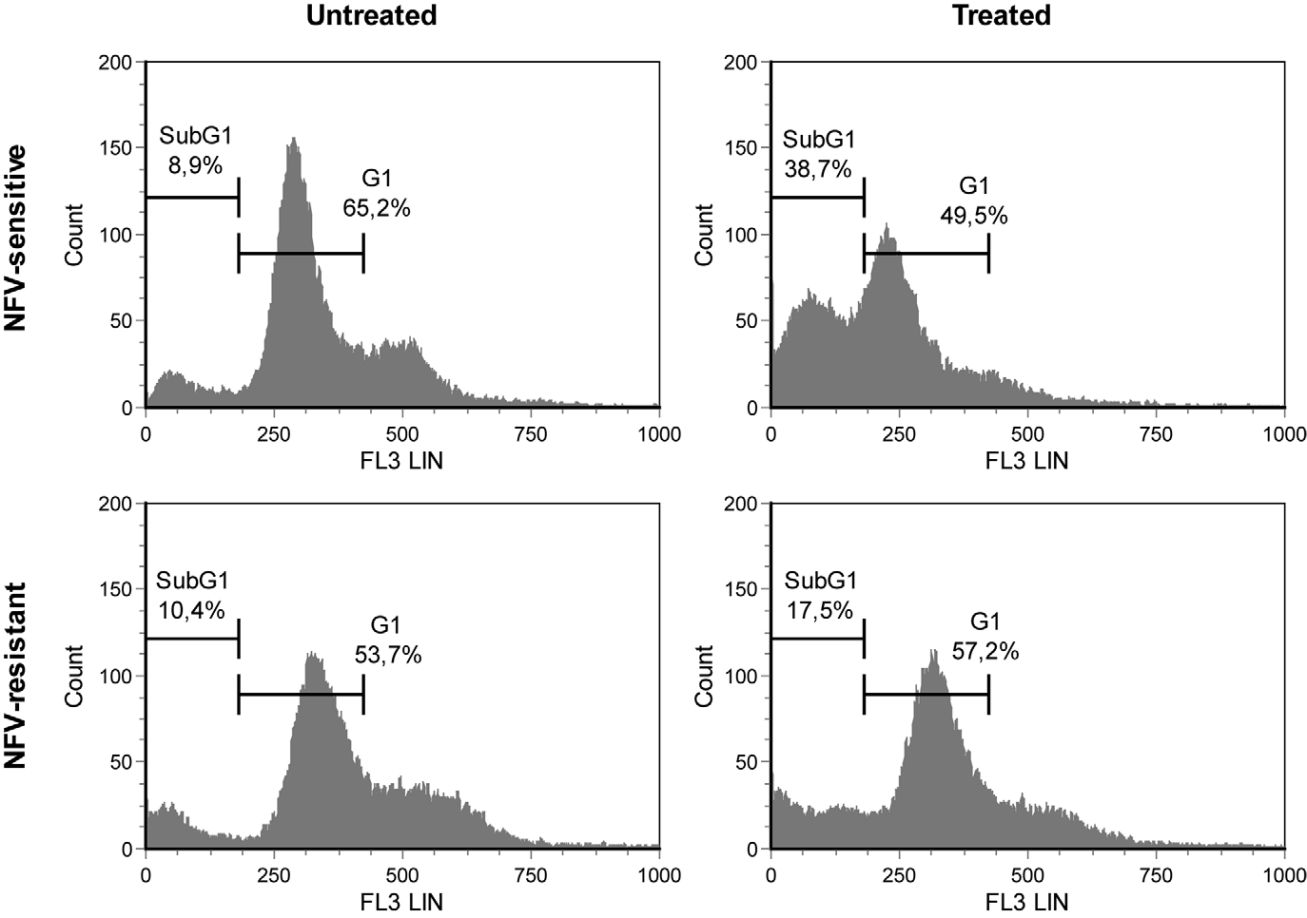
NFV induces an increase in the sub-G1 DNA-containing population. The DNA content degradation profiles of drug-sensitive and NFV-resistant axenic amastigotes (i.e. usually maintained under a constant drug pressure of 12.5 µM), which were either left untreated or treated with NFV (10 µM), were assessed by flow cytometry after cell permeabilisation and PI staining. DNA fragmentation was quantified by measuring the cell population in the sub-G1 DNA region indicated by M1 and G1 DNA peak indicated by M2. The data shown are representative of three independent experiments.

Next, to further confirm and evaluate the NFV-mediated induction of apoptosis, we performed an *in vitro* apoptosis detection assay by staining the sub-G1 DNA content of both NFV-sensitive (i.e. wild-type/WT) and -resistant Ld 9518 amastigotes (i.e. 12.5 µM) with propidium iodide. Next, the percentages of parasites containing sub-G1 DNA (below the G0/G1 peak) were measured by flow cytometry. After a 24 h treatment with NFV, the percentage of drug-sensitive amastigotes in the sub-G1 phase (i.e. M1 region) obtained was 38.7%, compared to 49.5% in the G1 fraction (i.e. M2 region) (Fig. 3). As expected, a low sub-G1 fraction is detected in untreated parasites (i.e. 8.9%), therefore confirming that NFV induces DNA fragmentation in axenic amastigotes after 24 h of treatment. Importantly, the percentage of parasites with a sub-G1 content are also less important in both untreated (i.e. 10.4%) and drug-treated NFV-resistant axenic amastigotes (i.e. 17.5%). Again, these observations are further suggesting that NFV causes an apoptosis-like death process in *Leishmania*.

### NFV induces a caspase-independent death process and oxidative stress in *Leishmania*

**Figure 4:**
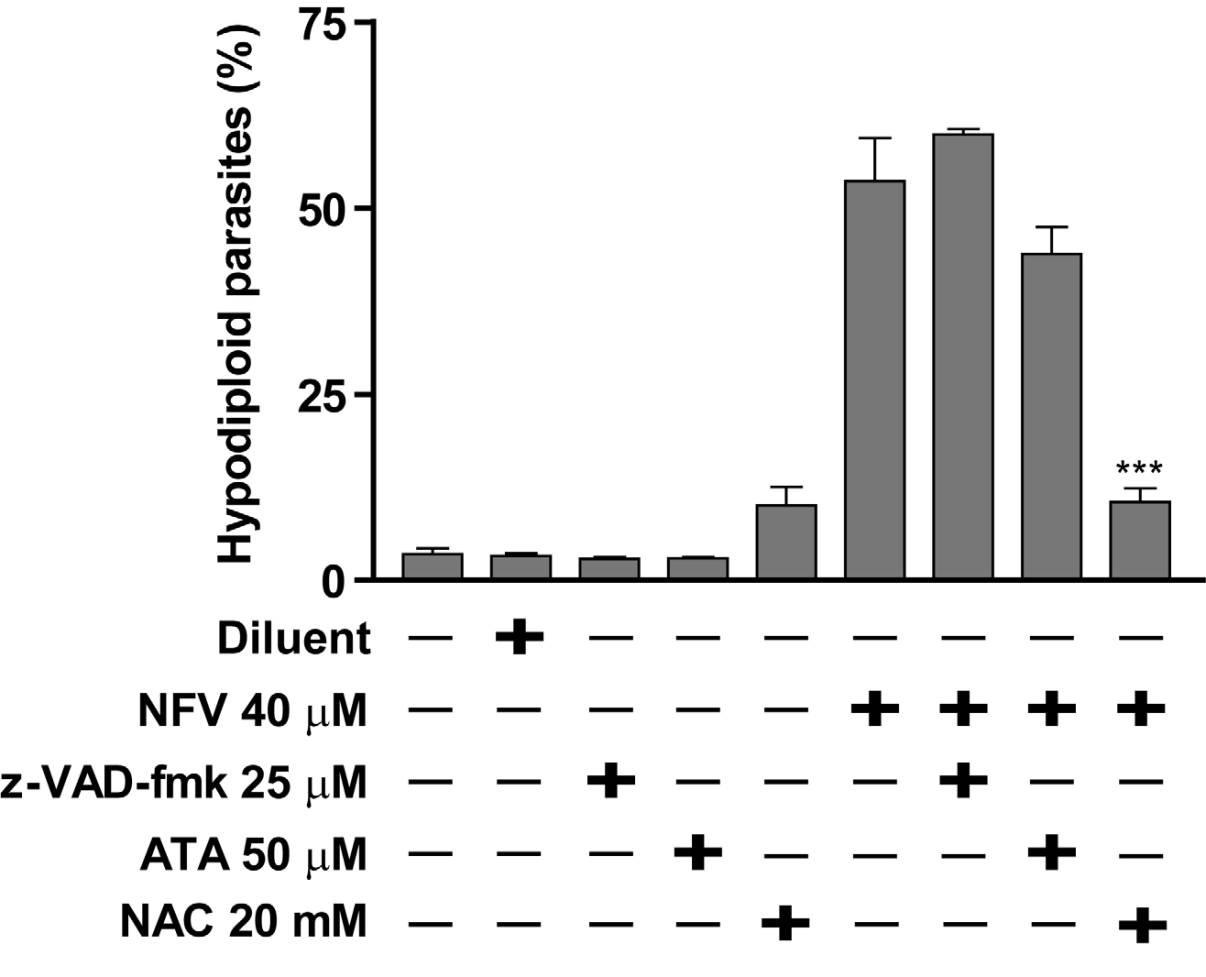
NFV generates an oxidative stress-mediated caspase-independent death process. Drug-sensitive axenic amastigotes were either left untreated or treated for 2 h with the indicated compounds, i.e. caspase inhibitor z-VAD-fmk (25 µM), Endo-G inhibitor ATA (50 µM) and antioxidant NAC (20 mM). Next, parasites were subjected to a treatment for 24 h with NFV (40 µM) or the diluent (i.e. DMSO). Percentage of apoptotic cells was measured by staining with propidium iodide as described in Materials and Methods. Data are expressed as mean ± SD of three independent experiments.

As apoptosis involves endonuclease-mediated DNA fragmentation and caspases activate the endonuclease responsible for cleaving cellular DNA, a pan-caspase inhibitor (i.e. z-VAD-fmk) was used to investigate if these two pathways are involved in NFV-induced apoptosis in *Leishmania*. Pretreatment of the parasites with z-VAD-fmk (25 µM) did not bring any decrease in the percentage of sub-G1 DNA content in amastigotes with respect to NFV treatment, as the percentage of sub-G1 DNA in NFV-treated parasites, either pre-treated with z-VAD-fmk (57%) or not (54%), were similar (*P*>0.05) (Fig. 4). Similar observations were obtained when using higher concentrations of z-VAD-fmk (i.e. up to 100 µM) (data not shown). Therefore, NFV induces apoptosis in *Leishmania* in a caspase-independent manner.

We next examined the role of endonucleases in NFV-mediated DNA degradation. In this regard, parasites were pretreated with the endonuclease inhibitor aurin tricarboxylic acid (ATA), prior to NFV. Treatment of these parasites with both ATA and NFV exhibited an 18% reduction in sub-G1 DNA content in comparison to amastigotes only treated with NFV (Fig. 4). However, statistical analysis showed that this reduction was not significant (*P*>0.05).

Finally, we determined whether NFV induces oxidative stress in *Leishmania*. To this end, parasites were treated with the antioxidant N-acetyl-cysteine (NAC) prior to NFV. Our results show that NAC pre-treatment caused a 80% reduction in the sub-G1 DNA induced by NFV (Fig. 4), a statistically significant decrease when compared to the amount of sub-G1 DNA in NFV-treated parasites (*P*<0.001). Pre-treatment with NAC would therefore inhibit the production of ROS potentially generated by NFV, protecting cells from oxidative stress.

### NFV leads to a loss of mitochondrial potential and translocation of EndoG

**Figure 5:**
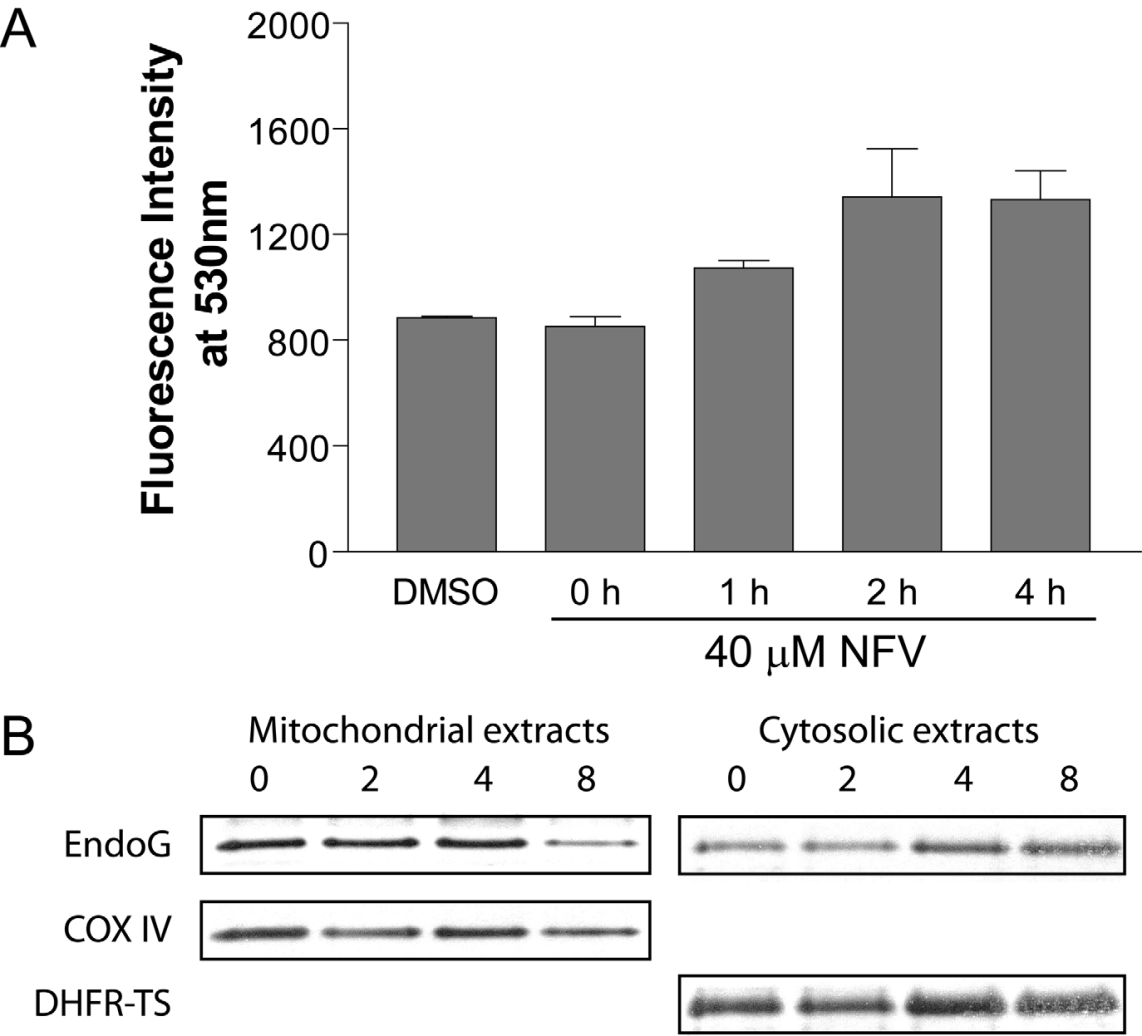
Loss of mitochondrial potential and translocation of EndoG in NFV-treated *L. donovani* amastigotes. (A) *L. donovani* axenic amastigotes were exposed to the diluent or NFV (40 µM) for the indicated time periods. Mitochondrial membrane potential (Δψ_m_) was assessed through the use of the JC-1 Mitochondrial Membrane Potential Detection Kit. Data are expressed as mean ± SD of three independent experiments. (B) Western blot analysis of the mitochondrial and cytosolic fractions obtained from NFV-treated amastigotes at different intervals. Anti-EndoG immunoblots of cytosolic and mitochondrial fractions are shown, along with the loading controls for mitochondria (i.e. COX IV) and cytosol (i.e. DHFR-TS), respectively.

A characteristic feature of apoptosis is the loss of mitochondrial potential (ψ_m_), which induces ROS formation and oxidative stress inside the parasite [22]. Using a dye (JC-1) sensitive to the loss in Δψ_m_, we monitored the mitochondrial membrane potential by spectrofluorometry. As shown in Fig. 5A, fluorescence increased with time upon NFV treatment. We observed a plateau in fluorescence intensity, corresponding to a complete loss of Δψ_m_, after 2 h following initial contact with NFV.

**Figure 6:**
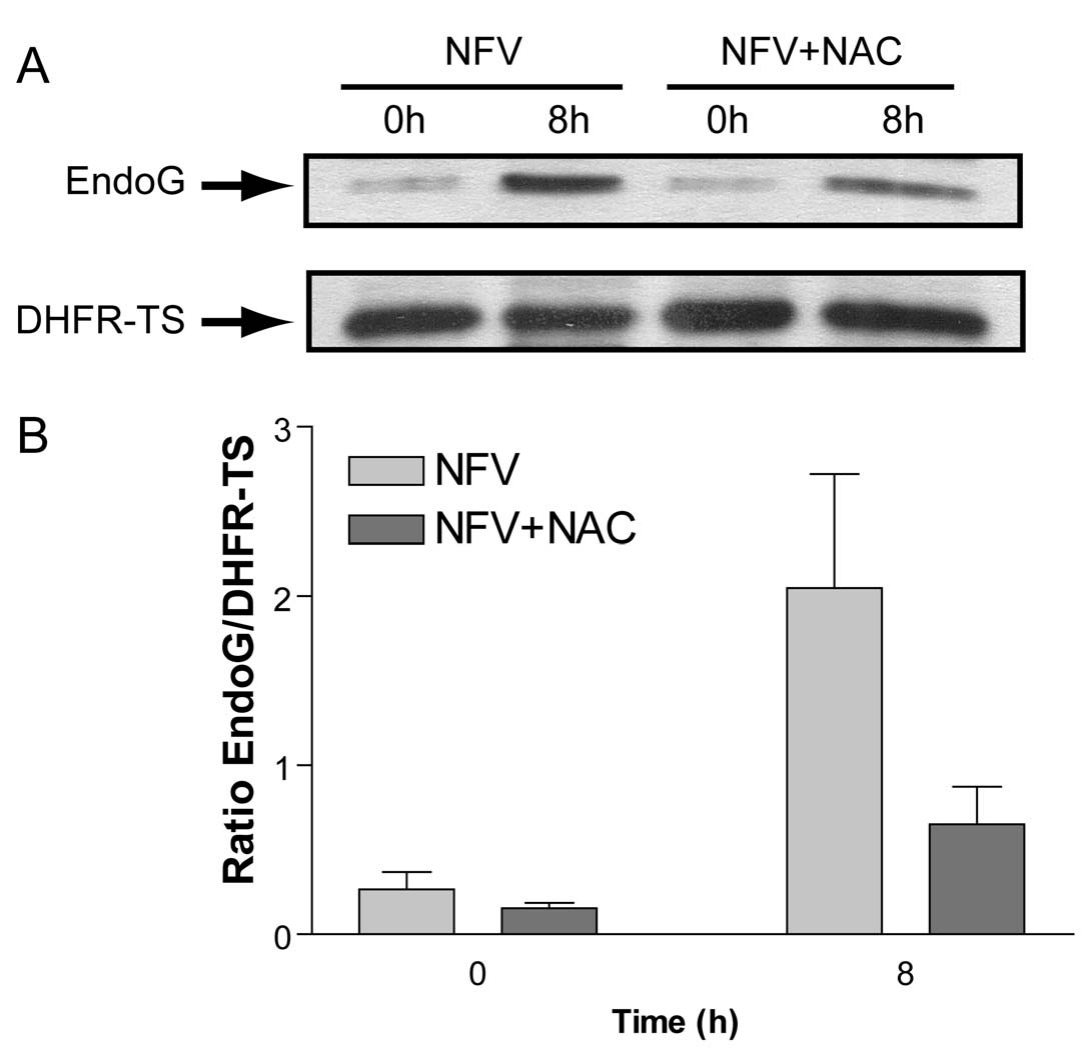
Cytosolic translocation of EndoG in NFV-treated *L. donovani* amastigotes is inhibited by addition of antioxidant. (A) *L. donovani* axenic amastigotes were exposed to NFV (40 µM) for 0 or 8 h with or without pre-treatment with NAC (20 mM). Western blot analysis of the cytosolic fractions obtained from the amastigotes was then performed using anti-EndoG (upper panel) and DHFR-TS (lower panel, as loading control). Densitometric analysis of blots from three of these experiments are shown in (B).

EndoG is involved in caspase-independent apoptosis and is a mitochondrial resident protein which cleaves the nuclear DNA [21],[31],[32], after its release from depolarized mitochondria through transition pores. Since our previous results suggested that NFV induces a loss of mitochondrial potential, we set out to determine if NFV also caused mitochondrial release of EndoG. Mitochondrial and cytosolic extracts prepared from both untreated and NFV-treated parasites were separated on SDS-PAGE, transferred to membranes and immunoblotted with anti-EndoG antibodies. The gradual translocation of EndoG to the cytoplasm was observed with time after NFV treatment (Fig. 5B). No variation in band intensities corresponding to COX IV (for the mitochondrial extracts) and DHFR-TS (for the cytosolic extracts) were observed, and were used as loading controls. Furthermore, this cytoplasmic translocation of EndoG was inhibited by the addition of NAC (Fig. 6).

### Expression of GSH1 protects amastigotes against NFV-induced cell death

**Figure 7:**
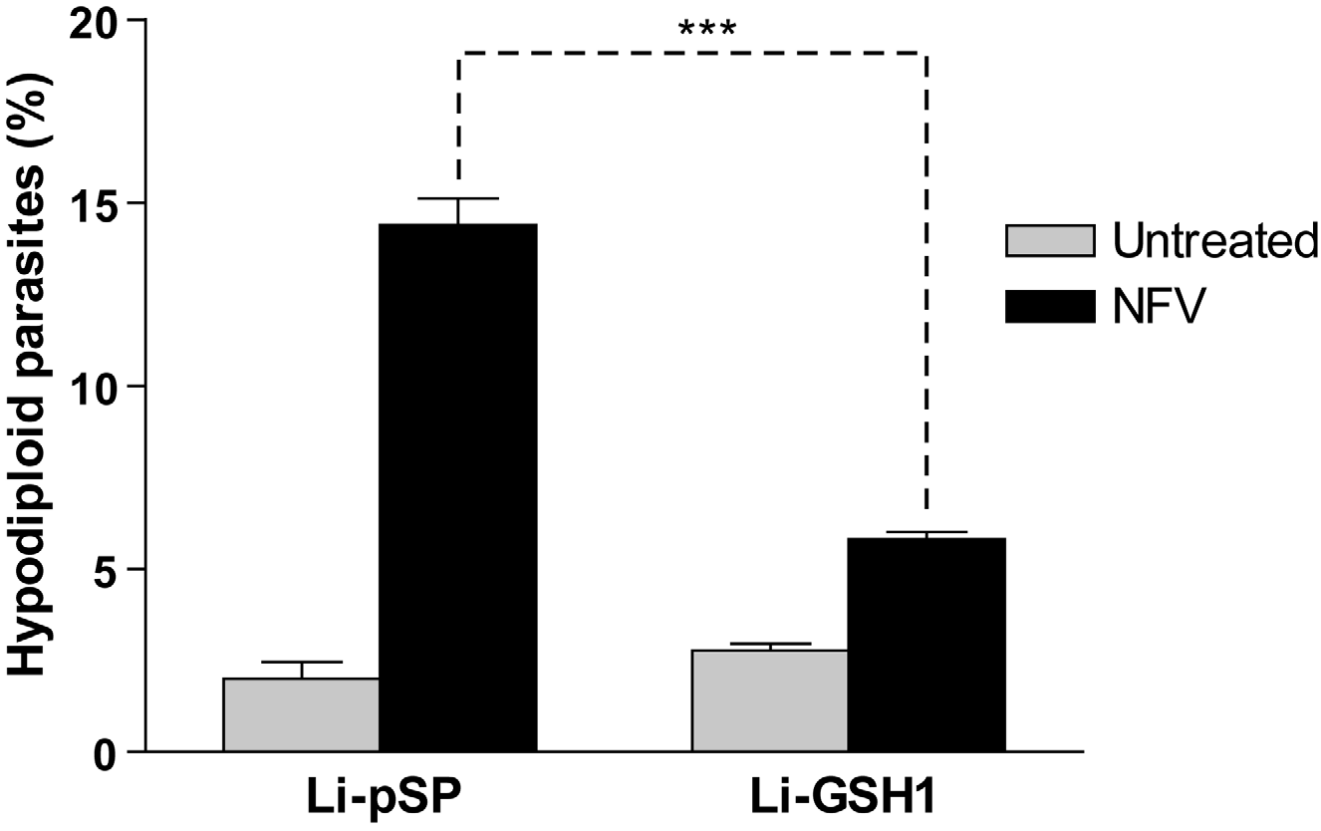
GSH1 overexpression suppresses NFV-induced cell death in parasites. Stably transfected *L. infantum* strains carrying either the empty control vector Pspαzeoα (i.e. Li-pSP) or the GSH1-encoding vector PspαzeoαGSH1 were exposed to NFV (40 µM) for 24 h. Cell death was measured in terms of DNA degradation. Light gray bars refer to untreated parasites, whereas black bars relate to NFV-treated parasites (*n* = 3) (***, *P*<0.001).

Glutathione (GSH) has been shown to protect cells from oxidative stress, as well as from the effect of several drugs. In order to further our understanding of the possible effect of NFV-induced ROS, we tested *L. infantum* axenic amastigotes transfected with the empty control vector pSPαzeoα (called Li-pSP) or a pSPαzeoα vector containing the *gsh1* gene (called Li-GSH1). The *gsh1* gene codes for the heavy subunit of γ-glutamylcysteine synthase (γ-GCS), the rate limiting enzyme of GSH biosynthesis in *Leishmania* [33],[34]. When Li-GSH1 parasites were subjected to a 12 h treatment with NFV, we observed a significant decrease (57%) in sub-G1 DNA content, when compared to NFV-treated control Li-pSP parasites (Fig. 7). This confirmed our previous observations that parasites generate ROS after exposure to NFV, and overexpression of GSH1 inhibits the effects of ROS in these cells.

## Discussion

In recent years, the number of worldwide reported cases of *Leishmania*/HIV-1 coinfections has been increasing steadily given the growing spread of the AIDS pandemic to smaller urban centers and more outlying urban and rural areas where *Leishmania* infections are more prevalent [1],[35]. Besides this expanding geographical overlap, these two diseases are changing their clinical, epidemiological and therapeutic aspects as they both impact on each other's development. For instance, in Africa and India, where *L. donovani*/HIV-1 coinfections are emerging, there are high rates of VL relapse and mortality [1],[36],[37]. Furthermore, the use of contaminated syringes by intravenous drug users has now also been identified as a way of transmission of *Leishmania*, as well as for HIV-1, particularly in Southern Europe and the Mediterranean basin [38]. In addition to these factors, *Leishmania* and HIV-1 act in a concerted manner in the coinfected individual, both benefiting from the other's presence. Indeed, *Leishmania* accelerates the progression toward AIDS, whereas HIV-1 can reinitiate or worsen *Leishmania*-related symptoms [2],[4],[5].

Improper diagnosis and the misuse of leishmaniacidal compounds have also complicated the treatment of *Leishmania*/HIV-1 coinfected individuals, and have contributed to the rise in the number of coinfected cases. For example, our group has previously reported that SbV, a widely used drug in the treatment against leishmaniasis, can accelerate HIV-1 replication under *in vitro* conditions [39]. These findings, combined with the fact that little is known on the effect of drugs designed specifically against HIV-1 or *Leishmania* in coinfected individuals, warrant further investigation into the impact of these compounds and their action on the opposite microbe.

In a recent paper, our group reported that drugs designed against the protease of HIV-1 markedly reduced the intracellular survival of *L. infantum* and *L. donovani* in both the macrophage cell line THP-1 and more physiologically relevant human monocyte-derived macrophages [11]. We also observed that, although we did not find any toxic effect of these drugs on promastigotes, the HIV-1 PIs NFV and RTV were effective against *Leishmania* axenic amastigotes at subcytotoxic levels. Given the long term potential of such findings for the treatment of *Leishmania*/HIV-1 coinfections, we further investigated by which mechanism HIV-1 PIs, specifically NFV, exert their effect on *Leishmania* amastigotes in culture. Besides being in the developmental stage of the parasite which is most affected by these drugs, it was also relevant to pursue such a study since amastigotes are the form of *Leishmania* usually found in humans. However, it should be pointed out that although the NFV concentrations used in this study were much stronger than those found in plasma of treated patients [40], it could be argued that, due to the accumulation of drug in macrophages [41], these higher concentrations may be relevant when used directly in media cultured parasites in order to compensate for this effect.

We first observed that nuclear DNA from NFV-treated axenic *L. donovani* amastigotes formed a long smear when resolved using agarose gel electrophoresis. Although not a classical ladder pattern associated with apoptosis, this degraded form of DNA is not uncommon in apoptotic *L. donovani*, as has been previously reported [18]. Similar patterns of DNA degradation have been observed in *Leishmania* parasites treated, among others, with MTF, a relatively recently developed antileishmanial drug which we have used in control experiments. Indeed, several other better established drugs are known to induce this effect on *Leishmania*, including pentavalant antimony, pentamidine, and amphotericin B [42]. However, our observations are of potential long term interest in the case of HIV-1/*Leishmania* coinfections, since NFV acts on both HIV-1 and the amastigote parasites *in vitro*.

The mechanisms involved in *Leishmania* apoptosis are not as well understood as in mammalian cells, but have been the object of several investigations in the recent past years [18],[21],[22],[43]. Indeed, some similarities can be made between both organisms. First, in both cases, mitochondria have been identified as major regulators of the apoptotic process, and permeabilization of the mitochondrial membrane by oxidative stress or other stresses leads to the release of proapoptotic enzymes in the cytosol. Relocation of cytochrome C and activation of cellular proteases act as irreversible commitments to the apoptotic death pathway. However, given that the equivalents of mammalian caspases have yet to be identified in *Leishmania*, at least part of the apoptotic machinery used by the parasite is caspase-independent. In this regard, we observed no effect of the caspase inhibitor z-VAD-fmk in NFV-induced apoptosis in *Leishmania*, suggesting that the mechanism(s) involved in this case is caspase-independent. Interestingly, Verma and colleagues reported that MTF activates caspase 3-like proteases in arsenite-resistant *L. donovani* promastigotes, and that their action was sensitive to the CED3/CPP32-specific inhibitor DEVD-fmk [44]. The recent identification of metacaspases in *Trypanosoma brucei* and *L. major* may further shed light on the specific pathways involved in *Leishmania* apoptotic cell death in response to drug treatments [43]. However, although metacaspases and caspases may be structurally similar, the catalytic activity of metacaspases in *Leishmania* is quite different from that of caspases, suggesting that different apoptotic pathways are involved.

Recently, several papers have proposed a role for EndoG, a mitochondrial protease, in chromatin degradation of trypanosomatids such as *Leishmania* and *Trypanosoma*. Gannavaram and co-workers showed that, when transfected with *L. major* EndoG, over-expressing *L. donovani* promastigotes exhibited increased sensitivity to oxidative stresses and engaged in translocation of EndoG to the cytosol [21]. Furthermore, Rico and colleagues demonstrated that *L. infantum* EndoG reacted similarly when these parasites are faced with apoptotic stimuli [23]. Finally, BoseDasgupta *et al.* further investigated the mechanisms of apoptosis in *L. donovani* promastigotes induced by the flavone baicalein, a topoisomerase-DNA complex stabilizer [22]. The authors found that, once released, EndoG formed a complex with flap endonuclease-1 (FEN-1), an enzyme involved in DNA repair, and, independently, with a Tat-D-like nuclease, in order to degrade DNA and possibly promote apoptosis. It is therefore possible that this is also the case in NFV-treated amastigotes, given that we also observed ATA-sensitive apoptosis and EndoG translocation to the cytosol. The same authors have also reported that the EndoG release from mitochondria is calcium-dependent and sensitive to the antioxidant NAC or the calcium chelator BAPTA-AM [22]. In this regard, we found that *L. donovani* axenic amastigotes responded in a similar, NAC-sensitive manner to NFV. Our finding that γ-GCS and GSH overexpression in *L. infantum* amastigotes, protecting the parasites from oxidative stress, counteracts the pro-apoptotic effects of NFV also suggests that EndoG is released in response to oxidative stress. It is therefore possible that very similar apoptotic pathways are activated in *Leishmania* promastigotes treated with baicalein, and amastigotes treated with NFV. However, additional experiments are warranted to clearly determine if such is the case.

With the expanding overlap between HIV-1 infection and leishmaniases in the world, it is now critical to better understand the multifaceted effects of antiviral and antiparasitic drugs in the case of HIV-1/*Leishmania* coinfected individuals. In this report, we determined how *L. donovani* amastigotes react to NFV, an HIV-1 PI, and that these processes lead to apoptotic death through a caspase-independent mechanism. Another group has recently described that HIV-1 PIs also affect *L. amazonensis* proliferation and infection of murine macrophages, and observed chromatin condensation in parasites suggesting apoptosis [13]. Further investigation will determine if such is a general reaction of trypanoplasmids to HIV-1 PIs, hopefully leading to a better understanding of these agents and possibly future therapeutic use in coinfected individuals. However, one must note that HIV-1 PIs may also exert toxicity in humans by inducing a mitochondrial loss of potential and apoptosis (for example, of T cells) [45],[46]. Therefore, careful evaluation of the benefits and pitfalls of such therapies must be evaluated when designing future chemotherapeutic strategies to test NFV as a potential possible antileishmanial agent as well as possibly in *Leishmania*/HIV-1 coinfections.
